# Novel multiplex assay platforms to detect influenza A hemagglutinin subtype‐specific antibody responses for high‐throughput and in‐field applications

**DOI:** 10.1111/irv.12449

**Published:** 2017-04-05

**Authors:** Zhu‐Nan Li, Jessica F. Trost, Kimberly M. Weber, Elizabeth H. LeMasters, Sharifa Nasreen, Javan Esfandiari, Angelo H. Gunasekera, Megan McCausland, Katharine Sturm‐Ramirez, Jens Wrammert, Sean Gregory, Vic Veguilla, James Stevens, Joseph D. Miller, Jacqueline M. Katz, Min Z. Levine

**Affiliations:** ^1^ Influenza DivisionNational Center for Immunization and Respiratory DiseasesCenters for Disease Control and PreventionAtlantaGAUSA; ^2^ Department of Microbiology and ImmunologyEmory UniversityAtlantaGAUSA; ^3^ Battelle Memorial InstituteColumbusOHUSA; ^4^ Centre for Communicable DiseasesThe International Centre for Diarrhoeal Disease Research, Bangladesh (icddr,b)DhakaBangladesh; ^5^ Chembio Diagnostic Systems, IncMedfordNYUSA

**Keywords:** antibody, Chembio Dual Path Platform, hemagglutinin, influenza, MAGPIX

## Abstract

**Background:**

Detections of influenza A subtype‐specific antibody responses are often complicated by the presence of cross‐reactive antibodies. We developed two novel multiplex platforms for antibody detection. The multiplexed magnetic fluorescence microsphere immunoassay (MAGPIX) is a high‐throughput laboratory‐based assay. Chembio Dual Path Platform (DPP) is a portable and rapid test that could be used in the field.

**Methods:**

Twelve recombinant globular head domain hemagglutinin (GH HA1) antigens from A(H1N1)pdm09 (pH1N1), A(H2N2), A(H3N2), A(H5N1), A(H7N9), A(H9N2), A(H13N9), B/Victoria lineage, B/Yamagata lineage viruses, and protein A control were used. Human sera from U.S. residents either vaccinated (with H5N1 or pH1N1) or infected with pH1N1 influenza viruses and sera from live bird market workers in Bangladesh (BDPW) were evaluated. GH HA1 antigens and serum adsorption using full ectodomain recombinant hemagglutinins from A(pH1N1) and A(H3N2) were introduced into the platforms to reduce cross‐reactivity.

**Results:**

Serum adsorption reduced cross‐reactivity to novel subtype HAs. Compared to traditional hemagglutination inhibition or microneutralization assays, when serum adsorption and the highest fold rise in signals were used to determine positivity, the correct subtype‐specific responses were identified in 86%‐100% of U.S. residents exposed to influenza antigens through vaccination or infection (N=49). For detection of H5N1‐specific antibodies in sera collected from BDPW, H5 sensitivity was 100% (six of six) for MAGPIX, 83% (five of six) for DPP, H5 specificity was 100% (15/15), and cross‐reactivity against other subtype was 0% (zero of six) for both platforms.

**Conclusion:**

MAGPIX and DPP platforms can be utilized for high‐throughput and in‐field detection of novel influenza virus infections.

## 1 Introduction

Hemagglutination inhibition (HI) and virus microneutralization (MN) assays are gold standards for detection of antibody responses to influenza; both tests primarily detect antibodies to hemagglutinin (HA) of influenza viruses.^1^, ^2^ However, implementation of these assays requires propagation of live viruses and appropriate biological safety levels, which often limits assay portability and throughput. Utilizing noninfectious recombinant proteins may broaden the applications of serologic assays, making them more suitable for field applications that are often at remote locations lacking heightened laboratory containment facilities.

Following novel influenza virus emergence events among humans such as the 2009 H1N1 pandemic, or H5N1 and H7N9 outbreaks, serologic studies need to be conducted quickly to assess the levels of exposure and immunity in populations. Rapid public health responses required in such scenarios highlight the need for sensitive and specific serologic assays to detect influenza subtype‐specific antibody responses that are quick, high throughput, and ideally portable.^2^, ^3^, ^4^

Given that the traditional ELISA often lacks subtype specificity^5^ and multiplexing capabilities^6^ to detect antibody responses to influenza, alternative assays continue to be evaluated to improve the performance of influenza serologic assays. A multiplex protein microarray using globular head domain HA1 (GH HA1) was developed to investigate antibody profiling, and high sensitivity and specificity were achieved by bivariate model of microarray using HAs from pH1N1 and A/South Carolina/1918 (H1N1) viruses.^4^, ^7^, ^8^ Recently, a mPLEX‐Flu assay using full‐length HA was also used to evaluate HA profiles and demonstrated good correlation between the median fluorescence intensity (MFI) and anti‐HA antibodies in ELISA.^9^

Detections of antibody responses to influenza in humans are often complicated by the complex antibody profiles generated from past exposure through infection or vaccination with multiple influenza viruses. Novel influenza viruses may share common epitopes with seasonal viruses that an individual may have been exposed to previously, causing both within‐subtype (homosubtypic) or cross‐subtype (heterosubtypic) cross‐reactivity. This cross‐reactive immunity may provide broader immune protection to influenza virus infection, but at the same time also poses challenges when serology is used to assess exposure with novel influenza viruses.

Here, we developed two novel multiplex assay platforms: the magnetic multiplexed fluorescence microsphere immunoassay (MAGPIX) and the Chembio Dual Path Platform (DPP) utilizing multiple subtype rHAs to address high‐throughput needs in the laboratory setting and the portability required for in‐field applications, respectively. We incorporated the use of GH HA1 as detection antigens and antibody adsorption using full ectodomain HAs to reduce the effects of antibody cross‐reactivity to improve assay performance. Panels of well‐characterized human serum specimens collected from persons exposed to known subtype influenza viruses or vaccines were used to assess assay sensitivity, specificity, and cross‐reactivity. In addition, sera from workers in Bangladesh live poultry markets that were potentially exposed to highly pathogenic H5N1 viruses were also evaluated to recapitulate the scenario in which these assays may be applied in the field in the event of novel influenza virus outbreaks.

## 2 Material and Methods

### 2.1 Human sera

Twenty‐one paired acute (S1, 1‐7 days post‐symptom onset) and convalescent (S2, 15‐52 days post‐symptom onset) sera were collected from A(H1N1) pdm09 (pH1N1) virus‐infected persons during the first wave of the 2009 H1N1 pandemic (April to July, 2009). All patients showed seroconversion by both HI and MN assays with convalescent HI and MN titers of ≥40. The collection of these sera did not require CDC Institutional Review Board (IRB) review because of CDC U.S. Public Health Emergency Response to the pandemic. Fifteen paired pre‐ (S1, day 0) and post‐vaccination (S2, day 28 or 56) sera were collected in 2009 from U.S. residents who received one dose of 15 μg pH1N1 monovalent, non‐adjuvanted, split vaccine. Paired sera were collected from 13 U.S. residents who received two doses of 3.75 μg A/Indonesia/05/2005 (H5N1) monovalent, ASO3‐adjuvanted split vaccine in 2013. Subjects that received pH1N1 or H5N1 vaccination showed seroconversions to vaccine antigens detected by either HI or MN assay, respectively. Human subject approval for use of the H5N1 vaccination sera was obtained from the CDC IRB; the use of pH1N1 vaccine sera was approved by Emory University IRB (Table 1). Paired serum samples were collected from live poultry market workers in Bangladesh as a part of a surveillance study to identify H5N1 infections. The persons were confirmed either seroconverted (six persons) or seronegative (15 persons) for antibodies against A/Bangladesh/3233/2011 (A/BD/3233/2011) (H5N1) by MN.^10^ Written informed consents were collected before enrollment, and the research protocol was approved by the IRB at the International Centre for Diarrhoeal Disease Research, Bangladesh (icddr,b) and CDC.

**Table 1:** Sera from influenza‐vaccinated or influenza‐infected persons used in the study for evaluation of sensitivity and cross‐reactivity

Serum source	Age range (yrs)	S1^a^	S2^b^	For sensitivity^c^		For cross‐reactivity^d^				
				pH1	H5	H2	H5	H7	H9	H13
H5N1 vaccinees^e^	30‐59	13	13	N/A	13	13	N/A	13	13	13
A(H1N1)pdm09 infected persons^f^	19‐49	21	21	21	N/A	21	21	21	21	21
pH1N1 vaccinees^g^	26‐64	15	15	15	N/A	15	15	15	15	15
Total number of specimens	19‐64	49	49	36	13	49	36	49	49	49

a Pre‐vaccination sera (S1) from vaccine studies or acute sera (S1) from A(H1N1)pdm09 virus‐infected persons were collected from US residents.

b Post‐vaccination sera (S2) or convalescent (S2) showed HI ≥ 40, MN ≥ 80, and fourfold or greater rise in antibody titer, all sera collected from US residents.

c Serum sample pairs were used to determine sensitivity by fold rises in MFI and DPP values for exposed antigens.

d Serum sample pairs were used to determine cross‐reactivity by fold rises in MFI and DPP values for unexposed antigens.

e Sera were collected from ASO3‐adjuvanted A/Indonesia/05/2005 (H5N1) vaccine study.

f Sera were collected from pH1N1‐infected persons.

g Sera were collected from split monovalent A/California/07/09 (pH1N1) vaccine study.

### 2.2 Serum adsorption assay

One milliliter of 1% blue latex beads (300 nm in diameter, Thermo Fisher Scientific, NY, USA) was conjugated with 50 μg of each full ectodomain rHA from A(H1N1)pdm09 (pH1N1) and A/Perth/16/2009 (H3N2) and lyophilized in one vial by Chembio Diagnostic Systems, Inc. (NY). One vial of lyophilized latex beads was reconstituted using 1 mL distilled water, and 10 μL of serum was diluted in 340 μL assay buffer (1× phosphate‐buffered saline (PBS) containing 1% bovine serum albumin [BSA, Sigma, MO, USA], 0.05% Tween 20 [Sigma], 0.5 mol L^−1^ NaCl [Sigma], 0.05% sodium azide) for MAGPIX and 440 μL diluent (50 mmol L^−1^ Tris‐HCl buffer, pH 8.0) for DPP followed by incubation with 50 μL of PBS (mock) or latex beads (adsorbed) at room temperature for 10 minutes. Both mock‐treated and latex beads adsorbed samples were filtered through a 0.2‐μm Mini‐UniPrep G2 syringeless filters (GE Healthcare, PA) for DPP or latex beads were removed by centrifugation at 9000 *g* for 15 minutes for MAGPIX. Mock‐treated or adsorbed serum samples were tested by MAGPIX and DPP platforms.

### 2.3 Magnetic multiplexed fluorescence microsphere immunoassay

Ten trimeric GH HA1 antigens from A/California/07/2009 (pH1N1), A/Japan/305/57 (H2N2), A/Texas/50/2012 (H3N2), A/Vietnam/1203/2004 (H5N1 VN, clade 1), A/Indonesia/5/2005 (H5N1 IN, clade 2.1.3.2), A/Shanghai/2/2013 (H7N9), A/Hong Kong/33982/2009 (H9N2), A/shorebird/Delaware/68/2004 (H13N9), B/Brisbane/60/2008 (B Victoria lineage, B/B), B/Wisconsin/1/2010 (B Yamagata lineage, B/W), and a protein A (PA) control were used in this study. The GH HA1 antigens were either obtained from the International Research Resource (https://www.internationalreagentresource.org/) or made in‐house using baculovirus‐infected insect cells at CDC as described previously.^11^, ^12^ Briefly, 60 μg of each GH HA1 or 22 μg of protein A (PA) was coupled to 6.25×10^6^ Bio‐Plex Pro^™^ Magnetic COOH beads (Bio‐Rad, CA, USA) following carbodiimide‐mediated peptide coupling protocol from Bio‐Rad (http://www.bio-rad.com/webroot/web/pdf/lsr/literature/4110012C.pdf). Fifty microliters of microspheres containing two thousand microspheres of each of eleven bead regions in assay buffer was added to each well of a black wall plate (BD, CA) (22,000 microspheres/well). Fifty microliters of mock or adsorbed serum samples (1:40) was incubated with rHA‐conjugated beads in the dark, at room temperature for 60 minutes with shaking. The plate was washed with 100 μL of assay buffer three times with Bio‐Plex Handheld Magnetic Washer (Bio‐Rad) followed by a 60‐minute incubation with 100 μL of PA‐phycoerythrin conjugate (protein A‐RPE) with shaking in the dark. The plate was washed three times with 100 μL of reading buffer (1× PBS with 0.05% Tween 20, 1% BSA, and 0.05% sodium azide) and then read by a Bio‐Plex^®^ MAGPIX^™^ Multiplex Reader. MFI was obtained and analyzed with Bio‐Plex Manager^™^ MP Software.

### 2.4 Chembio Dual Path Platform

The Chembio DPP kit (Chembio Diagnostic Systems, NY, USA) contains 7 GH HA1 antigen lines including combined pH1N1 and H3N2 (A/Perth/16/2009), H2N2, H5N1 (A/Indonesia/05/2005), H7N9, H9N2, H13N9, combined B/B and B/W, and PA control (Figure S1). Briefly, 80 μL of mock or adsorbed serum samples (1:50 diluted) was added to the sample port (Figure S1) to allow sera antibodies to bind to immobilized antigens on the membrane. After a 10‐minute incubation, five drops of running buffer were added to the buffer port (Figure S1) to allow colloidal gold‐conjugated PA to bind to GH HA1‐antibody complex for test line or to PA for control line, respectively. Results were read using a Chembio Rapid Influenza Immunity Test Reader (QIAGEN, Hilden, Germany) after 15 minutes of incubation at room temperature.

### 2.5 Data analysis

Fold rises in MFI and DPP values (S2 value/S1 value) were calculated to measure antibody binding. Due to wide dynamic ranges of the readout of the MAGPIX and DPP assays, S1 samples with values lower than baseline level were normalized to a set value as discussed previously.^13^ For serum samples from US residents, any MFI lower than 1000 in MAGPIX and DPP value lower than 100 in DPP in S1 samples were arbitrarily adjusted to 1000 and 100, respectively. Because the baseline antibody levels in S1 sera collected in Bangladesh were lower than those in S1 sera collected in the US (data not shown), low MFI and DPP values in Bangladesh S1 sera were arbitrary adjusted to 400 and 50, respectively. When fold rises against multiple subtype HAs (≥2) were observed, the subtype HA with the highest fold rise in MFI or DPP value was considered positive. Fisher exact test and paired sample *t* test were performed using GraphPad Prism 5 (GraphPad Software, Inc., La Jolla, CA, USA), and *P* values of <0.05 were considered statistically significant.

## 3 Results

### 3.1 Homosubtypic and heterosubtypic antibody responses following vaccination and natural infection are detected by MAGPIX and DPP

**Figure 1:**
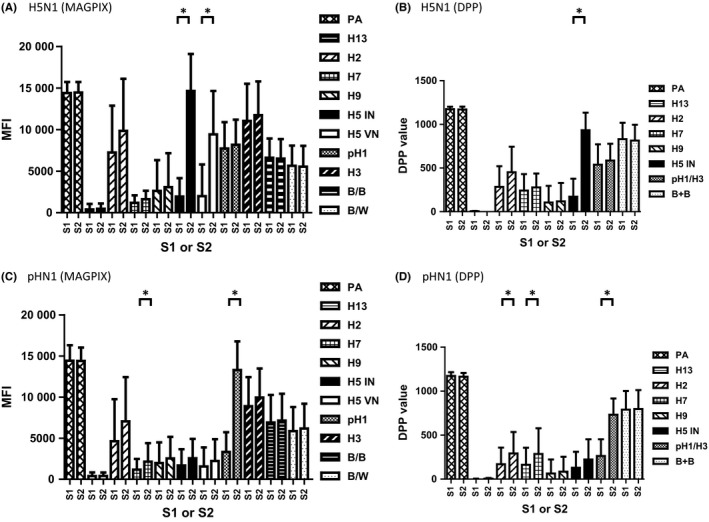
Increases in MFI and DPP values following vaccination and natural infection. Paired serum samples from ASO3‐adjuvanted split H5N1 vaccine study were tested by MAGPIX (A) and DPP (B). The paired serum samples from pH1N1‐exposed persons were tested by MAGPIX (C) and DPP (D). For each group, the mean MFI/DPP value and ± the standard deviation are shown.*P<.05

In untreated sera from persons vaccinated with a H5N1 (clade 2.1.3.2) vaccine, MFI and DPP values against H5 GH HA1 antigens rose significantly in MAGPIX and DPP, respectively (*P*<.05) (Figure 1A,B). Significant antibody responses against both H5 GH HA1 antigens (clade 1 and clade 2.1.3.2) were detected in MAGPIX (n=13, *P*<.05) (Figure 1A). Vaccination or infection with pH1N1 induced significant antibody responses to pH1 GH HA1 in MFI and to pH1/H3 in DPP (n=36, *P*<.05) (Figure 1C,D). It is noteworthy that in mock‐treated serum samples, heterosubtypic antibody responses against multiple non‐pH1 GH HA1 antigens were observed in both platforms (Figure 1C,D), although most were statistically not significant except MFI rise to H7 and DPP value rises to H2 and H7 in pH1N1‐exposed persons (n=36, *P*<.05) (Figure 1C,D).

### 3.2 Cross‐reactive antibodies can be removed by serum adsorption without loss of subtype‐specific antibodies

**Figure 2:**
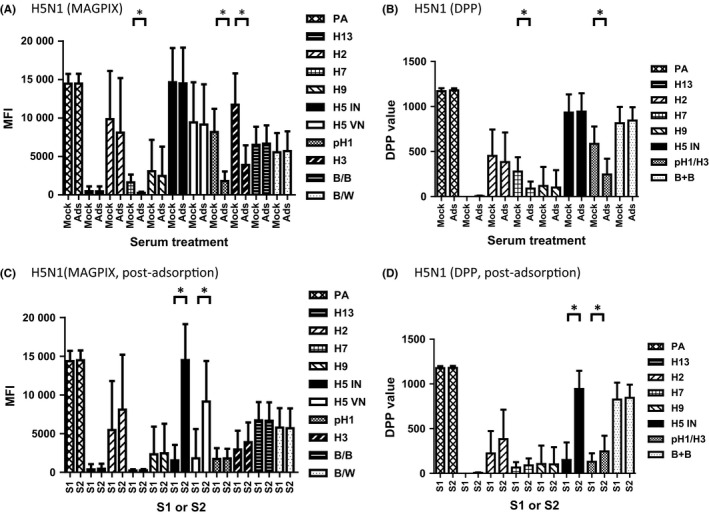
Cross‐reactive antibodies can be removed by serum adsorption without loss of subtype‐specific antibodies. Serum samples from persons who received ASO3‐adjuvanted split H5N1 vaccine were either mock or pH1/H3 rHA‐conjugated latex beads adsorbed. Pre‐ and post‐adsorption S2 sera were tested by MAGPIX (A) and DPP (B); post‐adsorption S1 and S2 sera were tested by MAGPIX (2C) and DPP (2D). For each group, the mean MFI/DPP value and ± the standard deviation are shown. *P<.05

To analyze subtype‐specific antibody responses due to antigen exposure, cross‐reactive antibodies were removed by serum adsorption with latex beads conjugated with ectodomain rHAs from pH1N1 and H3N2. Following adsorption of S2 sera from H5N1‐vaccinated persons (n=13), antibodies against pH1 and H3 in MAGPIX and pH1/H3 in DPP were significantly reduced (*P*<.05, Figure 2A,B); cross‐reactive antibodies to the novel subtype H7 GH HA1 were also removed (*P*<.05, Figure 2A,B). H5 GH HA1‐specific antibodies in H5N1 vaccinees remained post‐adsorption (*P*>.05, Figure 2A,B). This was also seen in paired serum samples from H5N1 vaccinees after serum adsorption (Figure 2C,D).

**Figure 3:**
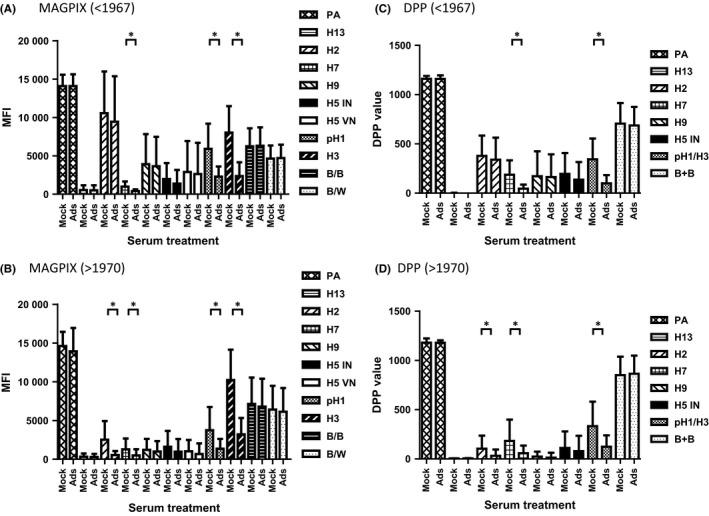
Higher anti‐H2 GH HA1 antibodies were detected in elderly population. The S1 serum samples were divided into two groups (born before 1967 and born after 1970), the S1 sera were adsorbed with pH1/H3 rHA‐conjugated latex beads, and mock‐treated or adsorbed sera were tested by MAGPIX (A and B) and DPP (C and D). For each group, the mean MFI and ± the standard deviation are shown. *P<.05. (A) <1967, the binding antibodies to H2 GH HA1 are consistent, *P*=.57. (B) >1970, the binding antibodies to H2 GH HA1 are reduced significantly, *P*<.05. (C) <1967, the binding antibodies to H2 GH HA1 are consistent, *P*=.60. (D) >1970, the binding antibodies to H2 GH HA1 are reduced significantly, *P*<.05

For the persons born before 1967 (n=17), antibody baselines (S1) against pH1, H2, H5 VN, and H9 were higher than those born after 1970 (n=32) in MAGPIX and DPP (*P*<.05). Antibodies to pH1, H3, and H7 GH HA1 antigens in S1 serum samples were removed by adsorption (*P*<.05, Figure 3). Of note, antibodies against H2 GH HA1 were removed by adsorption only in persons born after 1970 (n=32, *P*<.05, Figure 3), and not in persons born before 1967 (n=17, *P*>.05, Figure 3).

### 3.3 Improved sensitivity and reduced cross‐reactivity by serum adsorption for MAGPIX and DPP

S2/S1 fold rise threshold in MFI and DPP values was analyzed using paired human serum samples collected from either influenza infection or vaccination (Table 1). Some paired serum specimens showed a greater than twofold rise against multiple GH HA1 antigens due to cross‐reactivity or unknown exposure to avian influenza virus(es) (Table S1 and data not shown). Therefore, the highest fold rise value was used as a threshold to interpret results (Table 2). After serum adsorption with pH1/H3 rHA‐conjugated latex beads, the sensitivity for pH1/H3 in DPP increased from 67% to 86% (*P*=.052) (Table 2) and cross‐reactivity to H5 decreased from 11% to 3% (*P*=.357) (Table 2). The highest S2/S1 fold rise threshold post‐serum adsorption correlated well with the correct HA subtype‐specific antibody response when fold rises to multiple rHAs were observed (Table 2). 100% (MAGPIX) and 92% (DPP) of H5 vaccinees had the highest fold rise to H5 antigens, while 100% (MAGPIX and 86% (DPP) of persons either vaccinated or infected with pH1N1 had the highest fold rise to pH1N1 antigen.

**Table 2:** The highest fold rise in MFI or DPP values was used to determine the positivity

Serum source	Ads^a^	H5 (Indo)		pH1 (pH1 or pH1/H3)^b^	
		MAGPIX	DPP	MAGPIX	DPP
H5 vaccinees^c^	N	**100% (13/13)** d	**92% (12/13)**	0% (0/13)	0% (0/13)
H5 vaccinees^c^	Y	**100% (13/13)**	**92% (12/13)**	0% (0/13)	0% (0/13)
pH1N1 exposed persons^e^	N	3% (1/36)	11% (4/36)^f^	**97% (35/36)** d	**67% (24/36)** g
pH1N1 exposed persons^e^	Y	0% (0/36)	3% (1/36)^f^	**100% (36/36)**	**86% (31/36)** g

a Serum samples were either mock (N) or adsorbed (Y) with ectodomain pH1/H3 rHA‐conjugated latex beads.

b GH HA1 of pH1 and GH HA1 of pH1/H3 were used in MAGPIX and DPP, respectively.

c Paired sera were collected from 13 persons vaccinated with ASO3‐adjuvanted A/Indonesia/05/2005 (H5N1).

d Percent sensitivities for homologous HA subtype are highlighted in bold.

e Paired sera were collected from 36 persons infected with pH1N1 viruses or vaccinated with split monovalent A/California/07/2009 (pH1N1).

f *P*=.357.

g *P*=.052.

### 3.4 MAGPIX and DPP can detect antibody responses in poultry workers in Bangladesh with HPAI H5N1 exposure

To assess the performance of these platforms for in‐field applications in HPAI H5N1 virus surveillance or outbreak scenarios, sera collected from poultry workers in Bangladesh with confirmed seroconversion to a HPAI H5N1 virus A/Bangladesh/3233/2011 (H5N1) (n=6)^10^ and age‐matched sero‐negative poultry workers (n=15) were evaluated. When serum adsorption was performed and the highest fold rise in MFI and DPP values was used in analysis, assay sensitivity for H5 was 100% for MAGPLEX and 83% for DPP; specificity for H5 was 100% and cross‐reactivity was 0% for both platforms (Table 3). For mock‐treated samples, sensitivity was slightly lower (83% for MAGPIX and 67% for DPP) and only one person (one of six) showed cross‐reactivity against H2 GH HA1 (data not shown), although we could not exclude the possibility of potential exposure to H2 subtype avian influenza virus(es) for these poultry workers.

**Table 3:** The highest fold rise in MFI or DPP values was used to determine the sensitivity, cross‐reactivity, and specificity for Bangladesh poultry workers

Serum source	Ads^a^	Sensitivity^b^		Cross‐reactivity^c^		Specificity^d^	
		MAGPIX	DPP	MAGPIX	DPP	MAGPIX	DPP
Seroconversion^e^	N	83% (5/6)	67% (4/6)	0% (0/6)	0% (0/6)	na	na
Seroconversion^e^	Y	100% (6/6)	83% (5/6)	0% (0/6)	0% (0/6)	na	na
Seronegatives^f^	N	na	na	na	na	100% (15/15)	100% (15/15)
Seronegatives^f^	Y	na	na	na	na	100% (15/15)	100% (15/15)

a Serum samples were either mock (N) or adsorbed (Y) with ectodomain pH1/H3 rHA‐conjugated latex beads.

b Sensitivity was determined by the highest fold rise for H5 GH HA1 in MFI and DPP values (≥2 fold).

c Cross‐reactivity was determined by the highest fold rise for unexposed antigens in MFI and DPP values (≥2 fold).

d Specificity was determined by the highest fold rise for H5 GH HA1 in MFI and DPP values (≥2 fold).

e Sera were collected from H5N1‐infected persons that showed seroconversion to A/BD/3233/2011 (H5N1) in MN.

f Sera were collected from persons that showed seronegative to A/BD/3233/2011 (H5N1) in MN.

## 4 Discussion

Here, we developed and evaluated two novel multiplex platforms, MAGPIX and DPP, for the detection of subtype‐specific antibodies to influenza. We have improved assay sensitivity and specificity and reduced cross‐reactivity by two strategies. Firstly, GH HA1 antigens that contain more subtype‐ and strain‐specific epitopes were utilized as detection antigens to eliminate cross‐reactive epitopes in the HA2 domain.^14^ Secondly, serum adsorption with ectodomain rHAs from pH1 and H3 was introduced to remove cross‐reactive antibodies in human sera from past exposure to seasonal influenza viruses and/or vaccines. Real‐time stability studies indicated that there was only a 10% or lower decline in assay performance over 1‐year period under appropriate storage conditions for both platforms and rHA‐conjugated latex beads (data not shown).

In live poultry markets in Bangladesh where multiple subtypes and strains of avian influenza viruses cocirculate including highly pathogenic avian influenza (HPAI) H5N1 viruses, influenza surveillance among humans is needed to monitor the pandemic potential of novel avian influenza viruses. Although RT‐RCR is often considered as the standard method for influenza virus detection, viral RNA detection is limited to a short window of viral shedding. Extensive environmental contamination of viral material may pose challenges for diagnosis of influenza infection based on results only from RT‐PCR. In these scenarios, serological confirmation is important for accurate diagnosis of a true HPAI H5N1 infection in humans. However, serologic investigations may be hindered by the inability to perform traditional methods (HI or MN) in the field or lack of BSL3‐enhanced laboratories required for serologic assays with HPAI viruses. To simulate the use of these new assays in a real‐world situation, we utilized samples from humans that had been infected and vaccinated with multiple subtypes of viruses or had been exposed to novel influenza in Bangladesh poultry markets^10^, ^15^ to analyze both platforms. When sera were pre‐adsorbed with pH1/H3 rHA‐conjugated latex beads and the highest fold rise to antigen was considered as positive, >83% sensitivity (six of six or five of six), 100% specificity (15/15), and 0% cross‐reactivity (zero of six) were achieved for both platforms to detect exposures to H5N1 viruses (Table 3). These data indicated that these assays may have sufficient sensitivity and specificity to detect even mild or asymptomatic influenza virus infections.^10^ DPP is a portable lateral flow platform which is easy to use. Results from DPP can be obtained in a non‐laboratory setting in less than 30 minutes, making it an appealing choice for in‐field applications. MAGPIX is cost‐effective and high throughput, requires limited sample volume and staff time, and reduces aliquot errors.^6^, ^9^, ^16^, ^17^ Both platforms are multiplex assays with several influenza subtype antigens, ideal for serum specimen collected from locations where multiple subtypes of influenza viruses cocirculate. Our results suggested that the optimized DPP and MAGPIX platforms described here could be used for in‐field and high‐throughput applications to support both routine influenza surveillance and outbreak responses of HPAI influenza.

As a result of exposure(s) to seasonal influenza viruses and/or vaccine(s), antibodies to novel HA in unexposed populations increases with age and may vary by geographic location.^3^, ^18^, ^19^, ^20^, ^21^ People who were born before 1967 showed significantly higher reactivity against H2 GH HA1 than those born after 1970 (*P*<.05) and could not be removed by serum adsorption. They also showed higher antibody baselines (S1) against other group 1 HAs, such as, pH1, H5 VN, and H9 due to potential exposure to H2N2 virus and more seasonal H1N1 viruses. Therefore, age‐matched controls are necessary to determine baseline level of pre‐existing antibodies in different birth year cohorts.^13^, ^22^

A complex relationship between antibodies against seasonal and novel avian influenza viruses was observed in humans^23^ and experimentally infected chicken and ferrets.^8^ Cross‐reactive antibodies to novel subtype HA in ELISA was first reported by Murphy and colleagues, and antibodies against H8 HA were detected by ELISA, but not by HI in primary H1N1 or H3N2 influenza virus‐infected children.^5^ While the HI assay measures the presence of antibodies that inhibit HA binding to host cells, both MAGPIX and DPP assays were designed to detect total binding antibodies to GH HA1. In this study, the use of GH HA1 antigens improved the specificity of MAGPIX and DPP platforms for the detection of HA subtype‐specific antibody responses, although cross‐reactive antibodies against other subtype HA(s) were still observed without adsorption (Figure 1C,D).

We demonstrated that serum adsorption in combination with the use of GH HA1 reduced cross‐reactivity to other subtypes of GH HA1 without loss of subtype‐specific antibodies against H5 and H2 GH HA1 antigens (Figures 2 and 3). Serum adsorption improved sensitivity from 67% to 86% and reduced cross‐reactivity from 11% to 3% (Table 2). Upon exposure to one influenza antigen, antibody rises to multiple rHA subtypes were observed in both DPP and MAGPIX assays (Table S1), but the antigen with the highest fold rises can be used to identify correct subtype exposures in the majority of cases after serum adsorption (Table 2).

Our study has several limitations: (i) the availability of only small numbers of sera from persons with relevant exposures; (ii) sera were collected from different geographic locations and from populations with different demographics; (iii) unknown baseline levels in the general population from where sera were collected. (iv) For newly emerged viruses, there would also be a lead time required to produce rHAs which may delay the availability of the assay in an outbreak setting. However, this might not be critical for antigenically diverse H5 viruses, as similar antibody responses against both GH HA1 antigens from clade 1 and clade 2.1.3.2 were observed in H5N1‐exposed individuals that either received ASO3‐adjuvanted vaccines (Figures 1 and 2) or H5N1 virus‐infected (data not shown). (v) The assay may require appropriate selection of specific virus strain rHA to use in serum adsorption. (vi) When there are fold rises to multiple subtypes, although rare, we could not exclude the possibility of potential co‐infections.

In summary, portable lateral flow (DPP) and high‐throughput platform (MAGPIX) using GH HA1 proteins, combined with serum adsorption of ectodomain pH1/H3 rHAs, showed advantages in detecting HA subtype‐specific antibody responses. To our knowledge, this is the first study in which serum adsorption with rHA‐conjugated beads has been used to remove cross‐reactive antibodies in evaluating binding antibody assays. These platforms can be utilized in influenza serologic surveillance and outbreak responses to novel HPAI influenza virus infections, especially when the virus isolation and RT‐RCR results are not available or cannot be accurately interpreted without further serologic confirmation.

## Conflict of Interest

Coauthors Javan Esfandiari and Angelo H. Gunasekera are employed by Chembio Diagnostic Systems Inc. and hold company stocks. These coauthors did not have any additional role in the study design, data collection and analysis, decision to publish, or preparation of the manuscript. All other authors report no potential conflicts. Conflicts that the editors consider relevant to the content of the manuscript have been disclosed.
