# Supplementary material for: Novel multiplex assay platforms to detect influenza A hemagglutinin subtype‐specific antibody responses for high‐throughput and in‐field applications
Source: Influenza Other Respir Viruses. 2017 Apr 5;11(3):289–97. doi: 10.1111/irv.12449 (PMC5410722; doi:10.1111/irv.12449)
Supplement: Supplementary file 1 [file IRV-11-289-s001.pptx]

## Slide 1
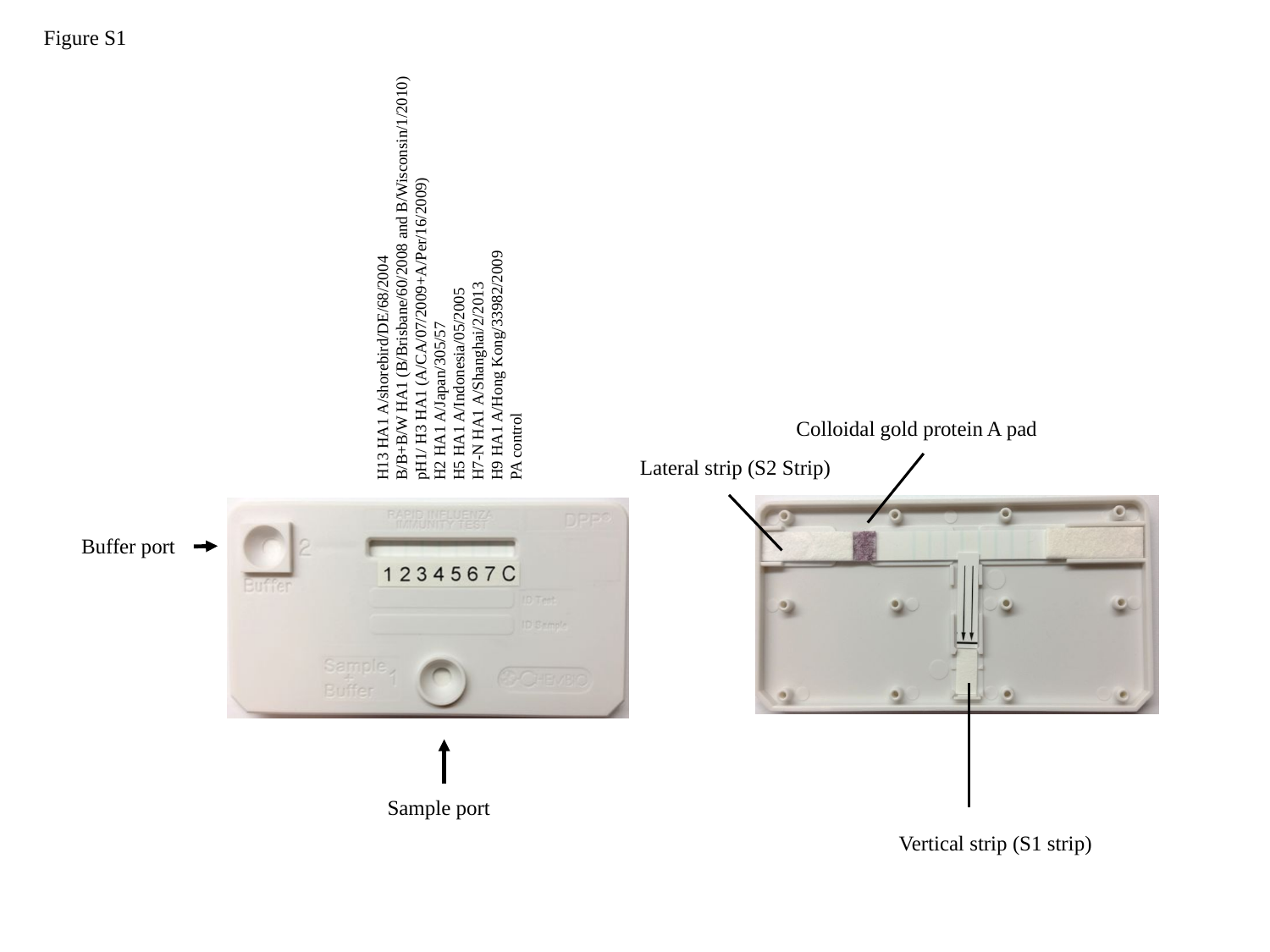

Figure S1
H13 HA1 A/shorebird/DE/68/2004
B/B+B/W HA1 (B/Brisbane/60/2008 and B/Wisconsin/1/2010)
pH1/ H3 HA1 (A/CA/07/2009+A/Per/16/2009)
H2 HA1 A/Japan/305/57
H5 HA1 A/Indonesia/05/2005
H7-N HA1 A/Shanghai/2/2013
H9 HA1 A/Hong Kong/33982/2009
PA control
Colloidal gold protein A pad
Lateral strip (S2 Strip)
Buffer port
Sample port
Vertical strip (S1 strip)
