# Supplementary material for: Novel multiplex assay platforms to detect influenza A hemagglutinin subtype‐specific antibody responses for high‐throughput and in‐field applications
Source: Influenza Other Respir Viruses. 2017 Apr 5;11(3):289–97. doi: 10.1111/irv.12449 (PMC5410722; doi:10.1111/irv.12449)
Supplement: Supplementary file 2 [file IRV-11-289-s002.docx]

| Table S1. Sensitivity and cross-reactivity for MAGPIX and DPP platforms when the threshold is at 2 fold rise from S1 to S2^a^ | | | | | | | | |
| --- | --- | --- | --- | --- | --- | --- | --- | --- |
| Platform | Sensitivity^b^ | |  | Cross-reactivity^c^ | | | | |
|  | pH1 | H5 |  | H2 | H5 | H7 | H9 | H13 |
| MAGPIX | 94% (34/36)^d^ | 100% (13/13)^e^ |  | 16% (8/49) | 5% (2/36) | 2% (1/49) | 2% (1/49) | 0% (0/49) |
| DPP | 81% (29/36)^d^ | 92% (12/13)^e^ |  | 18% (9/49) | 8% (3/36) | 10% (5/49) | 0% (0/49) | 0% (0/49) |
| ^a^ Sensitivity and cross-reactivity were determined by 2-fold rise in MFI and DPP values for mock treated samples | | | | | | | | |
| ^b^ Sensitivity was determined by using serum samples collected from either vaccinated or infected persons who showed serum conversion in HI and/or MN | | | | | | | | |
| ^c^Cross-reactivity was determined by 2-fold rise in MFI and DPP values for unexposed antigens. | | | | | | | | |
| ^d and e^ *p*>0.05 | | | | | | | | |
